# Latent Toxoplasmosis and Human

**Published:** 2012

**Authors:** A Dalimi, A Abdoli

**Affiliations:** Department of Parasitology, Faculty of Medical Sciences, Tarbiat Modares University, Tehran, Iran

**Keywords:** Toxoplasmosis, *Toxoplasma gondii*, Mental disorder, Behavioral parameter

## Abstract

Toxoplasmosis is one of the most common parasitic diseases worldwide. Although estimated that one third of the world's population are infected with *Toxoplasma gondii*, but the most common form of the disease is latent (asymptomatic). On the other hand, recent findings indicated that latent toxoplasmosis is not only unsafe for human, but also may play various roles in the etiology of different mental disorders. This paper reviews new findings about importance of latent toxoplasmosis (except in immunocompromised patients) in alterations of behavioral parameters and also its role in the etiology of schizophrenia and depressive disorders, obsessive–compulsive disorder, Alzheimer's diseases and Parkinson's disease, epilepsy, headache and or migraine, mental retardation and intelligence quotients, suicide attempt, risk of traffic accidents, sex ratio and some possible mechanisms of *T. gondii* that could contribute in the etiology of these alterations.

## Introduction

*Toxoplasma gondii* is an intracellular protozoan that infects approximately one-third of the world's population. Members of the cat family are final hosts of *T. gondii* and various warm-blooded animals including humans, are intermediate host. There are three infectious stages in the life cycle of *T. gondii*, including: oocysts containing sporozoites, tachyzoites and bradyzoites contained in tissue cysts (1).

Infection in human generally occurs through consuming food or drink contaminated with oocysts and tissue cysts from undercooked meat. Congenital transmission and organ transplantation are also other routes of infection (1).

Different conditions such as, number of parasite, virulence of the organism, genetic background, sex, and immunological status seem to affect the course of infection (1).

Most common form of the infections in humans are latent (asymptomatic) but in some conditions including immunocompromised patients and congenitally infected fetuses and newborns, may cause severe disease (2). Symptomatic infection is usually characterized by lymphadenopathy and reticular cell hyperplasia. Congenital infections acquired during the first trimester are more severe than those acquired in the second and third trimester, in congenital disease; hepatitis and pneumonia are followed by central nervous system (CNS) involvement resulting in hydrocephalus, retinochoroiditis and cerebral calcifications (3, 4). Ocular toxoplasmosis can be seen after congenital or acquired infection as a result of acute infection or reactivation (1). In immunocompromised patients such as AIDS, toxoplasmosis almost always happens as a result of reactivation of chronic infection. In these patients, clinical symptoms consist of mental status changes, seizures, sensory abnormalities, cerebellar signs, movement disorders, and neuropsychiatric findings (1).

*Toxoplasma gondii* consists of three main genotypes, designated type I, II, and III which differ in virulence and epidemiological pattern. Type I strain is associated with high-level virulence in mice (5). This type has been recorded in patients with ocular toxoplasmosis (6). Type II is nonvirulent for mice but generate chronic infection with persistence of tissue cysts. This strain is also most commonly associated with human infections in Europe and North America (5). Type III is nonvirulent for mice and less frequent than type II in Europe and North America. This Type is most frequent strain from animals (5). Moreover, Type I and II strains have been recorded in patients with congenital disease and AIDS patients (7, 8).

Although, seroprevalence rate of toxoplasmosis estimated between 20%-80% in different parts of the world including Iran (9, 10), but, the most common form of the disease is latent (asymptomatic). In fact after ingestion of the parasite and proliferation of tachyzoites in various organs during the acute stage, the parasite forms cysts in the brain and establishes a chronic infection in human and rat (11, 12). A variety of brain cells, including astrocytes and neurons, can be infected (13). So, the infection may causes various hormonal and mental disorders (14–17); moreover, other findings have shown that, latent toxoplasmosis can cause a wide range of behavioral changes in humans and animal models (18–21).

The purpose of this review article is demonstration of new finding on the impact of latent toxoplasmosis on human.

### Latent toxoplasmosis and behavioral parameters

Recent studies demonstrated that latent infection with *T. gondii* can alter behavioral parameters of human and rodents (18, 20). These alterations in rodents are named with the term of ‘parasite manipulation’ and in human by ‘parasite constraint’ (19). *Toxoplasma gondii* has only one final host (cat) but many intermediate hosts (all warm-blooded vertebrates). Rodents are persistent intermediate host reservoir for *T. gondii* in natural conditions; hence manipulation hypothesis mention a parasite may alter host behavior for its own benefit, usually by enhancing its transmission rate (19). Humans are dead-end hosts for *T. gondii*, because that rarely eaten by feline. In these conditions, induction of behavioral alterations termed as ‘parasitic constraint’ (19).

Different studies have been conducted on the relationship between latent toxoplasmosis and behavioral parameters in humans. Interestingly these parameters are different in infected men and women. For example, intelligence, affectothymia (warm, outgoing, attentive to others, kindly, easy-going, participating and likes people) and superego strength (rule-conscious, dutiful, conscientious, conforming, moralistic, staid and rule bound) are higher in infected women, while infected men have lower intelligence, superego strength and novelty seeking (low novelty seeking indicates rigid, loyal, stoic, slow-tempered and frugal personalities); both infected men and women have higher levels of guilt-proneness (they tend to be more apprehensive, self-doubting, worried, guilt prone, insecure, worrying and self-blaming) (22–27).

Flegr (2007) suggested, “Infected men were more likely to disregard rules and were more expedient, suspicious, jealous, and dogmatic and infected women more likely warm hearted, outgoing, conscientious, persistent, and moralistic”(20). Furthermore, other studies showed infection with *T. gondii* in humans can cause diminish in reaction time and psychomotor performance (28–30).

In animal models, *T. gondii* infected rats also observed significantly more active and less fear of novelty (neophobic fear) than uninfected animals (31, 32). Likewise, Hodcova et al. found learning capacity of *T. gondii* infected mice was diminished in static rod test and 8-arm radial maze test but spontaneous activity of these mice was increased in the wheel running test than non infected animals (33). Other studies showed the aversion of *T. gondii* infected rodents to predator odors (cat) were decreased compared to non infected animals, this event may cause increase predation risk of infected rodents and also increase transmission the parasite (34–36).

These observations support consents with the “behavioral manipulation” hypothesis, which states a parasite may alter host behavior for its own benefit by enhancing its transmission rate (19).

### Latent toxoplasmosis, schizophrenia and depressive disorders

Schizophrenia is a chronic, neuropsychiatric disease of uncertain cause that affects approximately 1% of people (37). Genetic and environmental factors including some infections (e.g. rubella, influenza, *T. gondii*, herpes simplex virus type 2 (HSV-2)) play roles in its etiology (38). In recent years, many studies have reported the higher incidence of *Toxoplasma* infection in schizophrenia patients. Majority of case control studies have been conducted on anti *T. gondii* antibodies test in schizophrenia patients; confirmed the higher infection with *T. gondii* (39–56). For example, 42.1% of patient with first-episode schizophrenia and 11.1% of healthy controls were seropositive to *T. gondii* infection (*P* < 0.007) (39).

The result of a meta-analysis of 42 studies which carried out in 17 countries showed the odds ratio of *T. gondii* antibodies in individuals with schizophrenia was OR 2.73 (57).

Another study demonstrated individuals with higher levels of *T. gondii* IgG antibodies significantly having more severe symptoms of psychoses (55). In addition, significant positive associations between increased levels of *T. gondii* IgG antibodies in schizophrenia patients were also observed with hazard ratio of 1.24 (58).

In addition infection with *T. gondii* may confer an increased risk of mortality in individuals with schizophrenia. Dickerson et al. have examined antibodies to *T. gondii* in 358 patients with schizophrenia and followed-up these peoples up to 5 years. The results indicated that the overall mortality rate was 8.6% for schizophrenia patients who were seropositive to *Toxoplasma* compared with 1.7% for seronegative schizophrenia patient (*P* < 0.003) (59).

There are also evidences that maternal and prenatal infection with *T. gondii* are as risk factors for schizophrenia and psychoses in adult offspring (60, 61). In this regard, Brown et al., have measured maternal anti-*T. gondii* IgG antibody in 63 women who their infants later developed schizophrenia or other schizophrenia spectrum disorders. Accordingly, the OR of schizophrenia and schizophrenia spectrum disorders increased in patients with high maternal anti-*T. gondii* IgG antibody (OR = 2.61; *P* = 0.051) (61). Another study in Denmark showed risk of schizophrenia in neonates with high levels of maternal anti-*T. gondii* IgG antibodies was significantly higher than control subjects (OR = 1.79; *P* = 0.045).

Recently, Horacek et al. found the gray matter density in the brain of schizophrenia patients who latently infected with *T. gondii* was significantly reduced than *Toxoplasma* negative schizophrenia patients in the caudate, median cingulate, thalamus and occipital cortex and in the left cerebellar hemispheres (62).

Furthermore, there are evidences that different genotypes of *T. gondii* have diverse effects on the course of psychosis. In this regard, Xiao et al. had investigated different genotypes of *T. gondii* in 219 pregnant women whose children developed schizophrenia and psychotic illnesses in adult life (63) and found that the risk for the development of psychoses in the offspring of mothers infected with genotype I *T. gondii* were significantly higher compared with the matched unaffected control mothers (*P* = 0.03).

The association of higher titers of *T. gondii* IgG antibody with anxiety, depression and schizophrenia spectrum disorders was also observed. Groër et al. observed higher titers of *T. gondii* IgG antibody was positively correlated with depression and anxiety in women during pregnancy. Indeed, depression and anxiety in pregnant women infected with genotype I *T. gondii* were highest than other genotypes; but this was not significant (64). Mothers with high titers of *T. gondii* IgG antibodies had significantly higher risk of schizophrenia spectrum disorders (65).

Some antischizophrenic and antipsychotic drugs can inhibit proliferation of *T. gondii* in vitro (66–68); interestingly, Kar and Misra reported a depressed patient with *Toxoplasma* seropositivity that showed poor response to anti-depressants drugs. The patient successfully treated with anti-depressants drugs only after treatment by anti-*Toxoplasma* drugs (pyrimethamine and sulphadiazine) (69).

### Latent toxoplasmosis and obsessive–compulsive disorder

Obsessive-Compulsive Disorder (OCD) is an anxiety disorder characterized by recurrent, unwanted thoughts (obsessions) and/or repetitive behaviors (compulsions). Repetitive behaviors such as hand washing, counting, checking, or cleaning are often performed with the hope of preventing obsessive thoughts or making them go away. OCD affects about 2.2 million American adults (70).

The rate of anti-*T. gondii* IgG antibodies among 42 patients with OCD and 100 healthy volunteers was studied and the seropositive rate among OCD patients was 47.62% significantly higher than the rate in healthy volunteers (19%; *P*<0.01) (71). Brynska et al. reported two children with symptoms of obsessive-compulsive disorder along with acquired toxoplasmosis. They found that OCD symptoms were observed immediately following acquired toxoplasmosis. These children were treated with anti-*Toxoplasma* drugs without any psy-chopharmacological treatment that caused significant improvement of OCD symptoms (72).

### Latent toxoplasmosis and suicide attempt

Suicide is the act of deliberately killing oneself. Risk factors for suicide include mental disorder (such as depression, personality disorder, alcohol dependence, or schizophrenia), and some physical illnesses, such as neurological disorders, cancer, and HIV infection. Global burden of suicide is one million deaths per year (73). In three independent studies, seroprevalence of toxoplasmosis in people who had suicide attempt were investigated (74–76). Arling et al. compared seropositivity and antibody titers of *T. gondii* between 99 patients with recurrent mood disorders with history of suicide attempt versus 119 patients with recurrent mood disorders without history of suicide attempt and 39 healthy controls individuals in US (74). Accordingly, no significant relation between *T. gondii* seropositivity and suicide attempt status was found; but the titers of *T. gondii* IgG antibodies in suicide attempters had significantly higher than non suicide attempters (*P* = 0.004). In addition, predictive association between history of suicide attempt and titers of *T. gondii* IgG antibodies was OR = 1.55, (*P*=0.006). Yagmur et al. have measured anti-*T. gondii* antibodies (IgG, IgM) in 200 cases of suicide attempts and 200 healthy volunteers in Turkey (75). As the result the positive rate for anti-*T. gondii* IgG antibodies was 41% and for IgM was 5.5% in suicide attempters, while the positive rate in controls were 28% and 5% for IgG and IgM antibodies. This differences for anti-*T.gondii* IgG antibody was statistically significant (*P*=0.004). In addition, there is a positive association between rates *T. gondii* infection and suicide in women of postmenopausal age (76).

### Latent toxoplasmosis and risk of traffic accidents

Traffic accidents are among the main reasons for public health damage. Nearly 1.3 million people die each year on the world's roads, and between 20 and 50 million sustain non-fatal injuries (77). In four studies, seroprevalence rate of toxoplasmosis in victims of traffic accidents and control group were investigated (78–81).

Flegr et al. in Prague (Czech Republic) found the risk of an accident in individuals with latent toxoplasmosis was 2.65 times higher than the toxoplasmosis-negative individuals (78). Moreover, the value of the odds ratio (OR) significantly increased with level of anti-*Toxoplasma* antibody titer (*P* < 0.0001); the OR of risk of an accident in subjects with low, moderate and high antibody titers were 1.86, 4.78 and 16.03 respectively.

The seroprevalence rate of *T. gondii* infection in victims of traffic accidents and in control group were 32.43% and 8.64% respectively (*P* <0.0001) in Turkey (79). In another study, the seroprevalence rate of toxoplasmosis was 53.5% for victims of traffic accidents and 28.0% in control groups (*P* <0.0001) (80). Furthermore, RhD molecule with unknown mechanisms has protective effect against traffic accidents in *Toxoplasma* positive individuals. In this study, 3890 male draftees were tested for *Toxoplasma* infection and RhD phenotype. The incidence rate of traffic accidents during their military service would be monitored. The result showed the probability of a traffic accident in RhD-negative individuals with high titers of anti-*Toxoplasma* antibodies was more than six times higher rate than *Toxoplasma* negative or RhD positive individuals (81).

### Latent toxoplasmosis, Alzheimer's diseases and Parkinson's disease

Alzheimer's disease is the most common neurodegenerative disorders that results in the irreversible loss of neurons, particularly in the cortex and hippocampus. Approximate prevalence of the disease is 1% among those 65 to 69 years of age, rising to 40 to 50 percent among persons 95 years of age and over. It is characterized clinically by progressive impairment in memory, judgment, decision making, orientation to physical surroundings, and language (82). Parkinson's disease is also the second most common neurodegenerative disorder, after Alzheimer's disease. Approximate prevalence of Parkinson's disease is 0.5 to 1 percent among persons 65 to 69 years of age, and increases with age to 1% to 3% among persons 80 years of age and older. The clinical manifestations of Parkinson's disease are characterized by resting tremor, bradykinesia, rigidity, and postural instability (82).

In a case control study about latent toxoplasmosis and Alzheimer's disease, the seropositive rate of anti-*T. gondii* IgG antibodies among individuals with Alzheimer's disease was 44.1% significantly higher those than control groups 24.3% (83). In addition, two studies evaluated the seroprevalence rate of toxoplasmosis in patients with Parkinson's disease.

The seroprevalence rate of anti-*T gondii* IgG antibodies in the patients with Parkinson's disease and control groups were 42.3 and 22.5% respectively, which is statistically significant (*P* = 0.006) (84). Although anti-*T. gondii* antibodies were detected in 50% and 40% of the patients with Parkinson's disease and in the control groups respectively, but the difference was not statistically significant (85).

### Latent toxoplasmosis, epilepsy, headache and or migraine

Epilepsy is the most common serious brain disorder worldwide affecting people of all ages, race and social class. It is characterized by recurrent seizures to severe and prolonged convulsions. The annual incidence rate of epilepsy is around 40–70 per 100,000 in industrialized countries and 100–190 per 100,000 in resource-poor countries (86). Several etiologic factors including infections (viral, bacterial, fungal and parasitic infection) in the etiology of epilepsy are involving (87).

In order to determine whether there is a relation between toxoplasmosis and epilepsy, Stommel et al. had measured anti-*T.gondii* IgG antibodies in 22 patients with cryptogenic epilepsy and 23 healthy controls. They found thta anti-*T.gondii* IgG antibodies among epileptic patients were significantly elevated compared to controls (59% increased in optical density, *P*=0.013) (88).

Also, in a case-control study 52% of cryptogenic epilepsy patients, 22% of known cause epilepsy patients and 18% of healthy controls were seropositive to *T. gondii*, the seropositive rate in cryptogenic epilepsy patients was statistically significant from other groups (*P* <0.01) (89). In contrast, there were not any associations between epilepsy and seropositive rates of *T. gondii* and *Toxocara canis* in 100 cryptogenic epileptic patients and 50 healthy volunteers (90).

The prevalence of anti-*T.gondii* IgG antibody in the migraine patient was statistically higher than control groups (91). The seropositive rate for patients with migraine was 44.2%, in healthy controls was 26.0%, and for control subjects with headache due to rhinosinusitis was 24%.

Palmer (2007) had performed a meta-analysis to clarify the association between latent toxoplasmosis and cryptogenic epilepsy. This study compared the seroprevalence of toxoplasmosis rates in 17 different countries, against the prevalence rates for epilepsy in those regions; and three case controlled studies about latent toxoplasmosis and cryptogenic epilepsy. The result showed that odds ratio of the three case controlled studies is 4.8, which indicated that epilepsy is 4.8 times more prevalent among *Toxoplasma* seropositive persons (92). Furthermore, the ecological study showed a strong association between the prevalence of epilepsy and the seroprevalence of toxoplasmosis within the same region (*P*<0.001). Palmer concludes, “The prevalence of toxoplasmosis is an important factor in the prevalence of epilepsy”. “An area with a reduced burden of toxoplasmosis will also have a reduced burden of epilepsy” and the higher seroprevalence of toxoplasmosis is associated with higher the rate of epilepsy in the population (92).

### Latent toxoplasmosis, mental retardation and intelligence quotients (IQ)

Mental retardation (MR) is one of the most common neuropsychiatric disorders among children and adolescents. Mental retardation is more frequent in males and its prevalence among young individuals is about 1%. Various factors including congenital infections are involved on its etiology (93). Prenatal infection with *Toxoplasma* and rubella probably responsible for about 2-3% of all the cases of mental deficiency, cytomegalovirus infection is also associated with about 10% of microcephalic mental deficiency (94).

A systematic investigation on the subject of latent toxoplasmosis and cognitive functions in *Toxoplasma* infected and non infected children (with comparison of social background, physical, behavior and intelligence quotients) showed an average IQ of infected children had lower than the controls (93 versus 110 respectively) (95). In addition, the results of a cases-control study among 450 mental retarded and 395 healthy children in Brazil displayed that 54.8% of the cases and 29.3% of controls were seropositive to *Toxoplasma*. Retinochoroiditis in mental retarded children was fourfold more prevalent than the healthy children and maternal exposure to cats and contact with soil were associated with an increase risk of mental retardation in this study (96). In this regard, Flegr et al. searched personality parameters of 857 *Toxoplasma* positive and negative military conscripts. They observed various personality parameters in the infected individuals were significantly lower than non infected controls. Moreover, they unexpectedly observed intelligence quotients (IQ) and educational levels of infected individuals were significantly lower than non infected individuals (25).

### Latent toxoplasmosis and sex ratio

The secondary sex ratio (the ratio of boy to girl at birth) is around 0.51 in most populations. This ratio may be influenced by various factors such as stress, immunosuppression, and age, the sex of preceding siblings, paternal endocrine disruption, and socioeconomic status of parents (97). A remarkable study in this regard showed that latent toxoplasmosis affect on the sex ratios in human. The results of a retrospective cohort study among 1,803 infants born from 1996 to 2004 in Czech Republic (with regarding the mother's age, concentration of anti-*Toxoplasma* antibodies, previous deliveries and abortion and the sex of the newborn) showed the secondary sex ratio in 454 *Toxoplasma* positive mothers was increased (proportion of males, 0.608) than in 1,349 *Toxoplasma* negative mothers (proportion of males, 0.527; *P*=0.0027), also after according of antibody concentration, the probability of the birth of a boy was increased with the increasing of titer of anti-*Toxoplasma* antibodies up to a value of 0.72 for 111 mothers with the highest titers; on the other word, “for every 260 boys born, 100 girls are born to women with the highest concentration of anti-*Toxoplasma* antibodies. Another study on mouse models reveal that mice with congenital toxoplasmosis in the early phase of latent infection produced a higher sex ratio and in the later phase of infection had a lower sex ratio than controls (98). Following the previous studies, Kaňková et al. suggested the immunosuppression effects of latent toxoplasmosis may increase sex ratio in mice and human. During latent toxoplasmosis in mice, significant modulation of the immune response and cytokine production occurred which it affect the secondary sex ratio (99).

### Some possible mechanisms of T. gondii that could contribute in the etiology of neurological and behavioral abnormalities

Brain is one of the most important locations for *T. gondii* cysts formation. A variety of brain cells, including astrocytes and neurons, can be infected (13). Various alterations such as anatomical, immunological, pathological, neurotransmitter and genes expression may occur during infection of brain with *T. gondii* (4, 100–102). Some of these alterations may also play roles in etiology of various neurological disorders.

Different neurological diseases of humans including schizophrenia, depression, Alzheimer's diseases and Parkinson diseases are associated with hippocampus and amygdala abnormalities (103–109). The hippocampus is a bilateral incurved seahorse-shaped structure of the cerebral cortex. It is involved in physiopathological processes of higher functions, like learning, memory, consciousness and information processing, language (110). The amygdala is also an almond-shaped group of nuclei at the heart of the telencephalon. It is associated with a range of cognitive functions, including emotion, learning, memory, attention and perception (111). There are evidences that *Toxoplasma* infection have greatest impacts on the hippocampus and amygdala. Vyas et al. studied bioluminescence imaging of the brain of rats infected with *T. gondii* (35). They found the density of tissue cyst was higher in amygdala region. Hermes et al. also investigated the effects of *T. gondii* infection on various neurological and behavioral abnormalities in chronically infected mice (101) and observed various factors such as inflammations and pathological findings were more common in hippocampus areas. The weight of brain in chronically infected mice was also decreased; moreover, in a magnetic resonance imaging (MRI) study a mild to moderate ventricular dilatation of the brain of chronically infected mice was revealed (101).

Another likely hypothesis which *T. gondii* could cause neurodegenerative and psychiatric disorders, is modulation of different neurotransmitters especially dopamine in brain by the parasite (112–114). The *T. gondii* genome is known to contain 2 aromatic amino acid hydroxylases that potentially could directly affect dopamine and/or serotonin biosynthesis. Dopamine is an important neurotransmitter which plays various roles in etiology of neuropsychological disorders including schizophrenia and other neurological diseases such as depression, Alzheimer's disease and Parkinson disease (112). Dopamine levels are also often increased in schizophrenia patients (112) and decreased in patients with Alzheimer's disease, Parkinson's disease and major depression (113). In this regards, serotonin or 5-hydroxy-3-indole acetic acid, norepinephrine, homovanillic acid and dopamine in the brain of acute and chronic *Toxoplasma* infected mice were measured so dopamine level was 14% increased in chronic infection but it was normal in acute infection. Homovanillic acid was 40% raised in acute but not in chronic infection. Norepinephrine was 28% decreased in acute but not in chronic infection. Although serotonin is implicated in a broad range of serotonin disorders like depression, schizophrenia, and Parkinson's disease, but it was not changed in acute and chronic infected mice (102). Treatment with a dopamine reuptake inhibitor (GBR 12909) alters the behavior of the mice infected with *T. gondii* (115). Treatment of infected rats with the haloperidol (a typical antipsychotic known dopamine D2 antagonist) and valproic acid (a mood-stabilizing drug use for treatment of epilepsy and bipolar disorder, but the mechanisms of its actions not fully understood) can reversed loss of fear to predator odor (116). Furthermore, *T. gondii* has two genes encoding tyrosine hydroxylase which that produces _L_-DOPA (117). This finding helps to better understand about dopamine alterations within the brain infected with *T. gondii*.

There are also evidences that inflammatory responses to infections may contribute to developing schizophrenia and related psychotic disorders (118, 119). Likewise, inflammatory responses are the innate defense against *Toxoplasma* infection (120).

Increased maternal levels of the inflammatory cytokine IL-8 and TNFα during pregnancy are associated with an increased risk for schizophrenia and psychotic illness among offspring (121, 122). Elevated IL-8 levels in pregnant women with anti-*T gondii* IgM antibody were also reported (123). IL-8 is responsible for activation and recirculation of neutrophils and increase during early infection with *T. gondii* (124).

Several reports described increased levels of IL-6, IL-2, and IL8 in schizophrenia patients (125–129). IL-6 is also associated with clinical feature of schizophrenia (127). Decrease levels of IL-2 and IFNγ were reported in schizophrenia patients during treatment with anti psychotic drugs (130, 131). IL-6 have protective role during early infection with *T. gondii* and IL-6 deficient mice are more susceptible for *T. gondii* infection (132). IL-6 is also a marker of ocular toxoplasmosis (133). There is evidence that *Toxoplasma* infected astrocytes and microglial cells release of IL-1α, IL-6 and granulocyte/macrophage colony-stimulating factor (GM-CSF) (134). On the other hand, IL-6 may mediate the exacerbation of autoimmune disorders in the CNS; in addition, there is strong association between IL-6 and neurotransmitter production (135). Furthermore, in vitro stimulation of neurons to secrete dopamine and probably other catecholamines by IL-6 were also reported (136).

Another possible correlation between *T. gondii* infection and schizophrenia is tryptophan metabolism. More than 95% of _L_-tryptophan in mammals is degraded through the kynurenine pathway. The two enzymes are capable of catalyzing the first step in the pathway, tryptophan 2, 3-dioxygenase (TDO) and indoleamine dioxygenase (IDO), which regulate of kynurenine metabolism (137, 138). The activity of IDO is mainly stimulated by IFN-γ and IL-2 (137). Kynurenic acid (KYNA) is an endogenous antagonist of both N-methyl-D-aspartate (NMDA) and α7 nicotinic acetylcholine (α7nACh) receptors. These two receptors are widely are involved in physiological processes underlying learning, memory and cognitive processes. The activities of both TDO and IDO in the brain are normally very low; but in some pathophysiological conditions like inflammation, activities of these receptors may increase that result high levels of KYNA. High levels of KYNA may contribute to the patients’ cognitive impairment (138–139). KYNA elevates in CSF of schizophrenia Patients (140–142).

On the other hand, tryptophan is an essential amino acid for *T. gondii* replication (143); and degradation of intracellular tryptophan by IDO which it mediated by IFN-γ inhibit intracellular replication of *T. gondii* (143, 144).

Astrocytes play a pivotal role in the production of KYNA in the CNS, because astrocytes are the main source of KYNA (119). Likewise, astrocytes are one of the most important cells that invade by *T. gondii* (145); astrocytes degraded tryptophan by IDO to inhibit *T. gondii* replication (145). Although, there are no any direct evidences which whether *T. gondii* infected cells produce KYNA after degradation of tryptophan by IDO but it is plausible.

## Conclusions

In this review article, new aspects of the latent toxoplasmosis discussed. About one third of the world's populations are latently infected with *T. gondii* without any clinical symptoms; however, recent studies indicated that latent toxoplasmosis may be playing various roles in the etiology of different mental disorders. The pathphysiology of mental illnesses originated from the brain dysfunction. Because the brain is one of the most important sites for *T. gondii* cysts formation, hence various alterations in brain may occur during infection. Some of these alterations may also play roles in etiology of various mental disorders. Until now, different hypotheses proposed that *Toxoplasma* infection involve in pathphysiology of mental illnesses. The role of *T. gondii* infection in hippocampus and amygdala abnormalities, neurotransmitters alterations (particularly dopamine), inflammatory responses in brain, tryptophan metabolism and kynurenic acid formation are the most of these hypotheses.
